# Chimeric padlock and iLock probes for increased efficiency of targeted RNA detection

**DOI:** 10.1261/rna.066753.118

**Published:** 2019-01

**Authors:** Tomasz Krzywkowski, Malte Kühnemund, Mats Nilsson

**Affiliations:** Science for Life Laboratory, Department of Biochemistry and Biophysics, Stockholm University, SE 171 65, Solna, Sweden

**Keywords:** chimeric oligonucleotides, invader, iLock, PBCV-1 DNA ligase, T4 RNA ligase 2

## Abstract

Many approaches exist to detect RNA using complementary oligonucleotides. DNA ligation-based techniques can improve discrimination of subtle sequence variations, but they have been difficult to implement for direct RNA analysis due to the infidelity and inefficiency of most DNA ligases on RNA. In this report, we have systematically studied if ribonucleotide substitutions in padlock probes can provide higher catalytic efficiencies for Chlorella virus DNA ligase (PBCV-1 DNA ligase) and T4 RNA ligase 2 (T4Rnl2) on RNA. We provide broad characterization of end-joining fidelity for both enzymes in RNA-templated 3′-OH RNA/5′-pDNA chimeric probe ligation. Both ligases showed increased ligation efficiency toward chimeric substrates on RNA. However, end-joining fidelity of PBCV-1 DNA ligase remained poor, while T4Rnl2 showed a somewhat better end-joining fidelity compared to PBCV-1 DNA ligase. The recently presented invader padlock (iLock) probes overcome the poor end-joining fidelity of PBCV-1 DNA ligase by the requirement of target-dependent 5′ flap removal prior to ligation. Here we show that two particular ribonucleotide substitutions greatly improve the activation and ligation rate of chimeric iLock probes on RNA. We characterized the end-joining efficiency and fidelity of PBCV-1 DNA ligase and T4Rnl2 with chimeric iLock probes on RNA and found that both enzymes exhibit high ligation fidelities for single nucleotide polymorphisms on RNA. Finally, we applied the chimeric probe concept to directly differentiate between human and mouse *ACTB* mRNA in situ, demonstrating chimeric padlock and iLock probes as superior to their DNA equivalents.

## INTRODUCTION

There is a large number of direct RNA analysis methods available. When a limited number of different targets are detected, fluorophore-conjugated complementary probes can be used in situ (Levsky and Singer 2003) and in vitro (Schena et al. 1995). Single mRNA molecules can be observed and quantified, or in parallel mRNA microarray format, readout signal intensity is proportional to RNA abundance allowing for comparative, quantitative mRNA expression assessment. However, hybridization-based methods are often limited by relatively low signal intensity, or poor hybridization specificity. To remedy this, DNA scaffolds with multiple detection sites can be sandwiched on target mRNA to increase signal intensity in situ (Player et al. 2001; Choi et al. 2014). Additionally, careful design of probe oligonucleotide lengths and predicted binding stability, limits formation of unspecific probe/target duplexes. However, optimal designs often have to be established empirically for each target. Despite continuous development of labeling chemistry and readout formats, methods offering robust single-nucleotide resolution sensing of RNA are not available.

Enzyme-assisted nucleic acid analysis methods are often superior over hybridization-based due to the high fidelity of enzymatic reactions. However, most of them require RNA to cDNA conversion before analysis. For example, DNA ligation assays perform much better on DNA substrates than on RNA substrates (Nilsson et al. 2000). Most DNA ligases do not tolerate RNA templates, and the ones that do, for example, Chlorella virus DNA ligase (PBCV-1 DNA ligase), have poor end-joining fidelity (Krzywkowski and Nilsson 2017). We have recently improved the end-joining fidelity of PBCV-1 ligase on RNA by adopting the highly specific nucleic acid sensing property of *Taq* DNA polymerase. In the iLock probe RNA assay, padlock probes (PLPs) with 5′ noncomplementary flaps (iLock probes) are activated by 5′ flap removal using the invader mechanism, provided correct base-pairing between probe and RNA target (Krzywkowski and Nilsson 2017). Although satisfactory sequence discrimination was achieved, RNA detection sensitivity was limited, especially for short target sequences such as miRNAs.

T4 RNA Ligase 2 (T4Rnl2) has been shown to have much higher end-joining activity for 3′-RNA/5′-DNA chimeric substrates (Nandakumar et al. 2004; Bullard and Bowater 2006), which motivated us to investigate if detection efficiency on RNA targets can be increased by combining chimeric RNA/DNA PLPs and rolling circle amplification (RCA) (Banér et al. 1998). While most DNA ligases have a very strict substrate specificity for nicks in DNA duplexes, a repertoire of ligases encoded by T4 bacteriophage genome, bacterial DraRN1 or viral PBCV-1 DNA ligase are able to join a diverse array of substrates on DNA or RNA (Bullard and Bowater 2006). T4 RNA Ligase 1 is commonly used as a template-independent RNA and DNA end-joining enzyme. T4Rnl2 is able to seal chimeric nicks composed of 3′-OH RNA and 5′-phosphorylated DNA on either DNA or RNA, and has mostly been used for small RNA next-generation sequencing library preparation (Bullard and Bowater 2006). Though PBCV-1 DNA ligase has been reported to seal DNA templated chimeric nicks (Sriskanda and Shuman 1998), RNA templated end-joining activity and fidelity have so far only been characterized for nonchimeric substrates in T4 DNA ligase and PBCV-1 ligase (Nilsson et al. 2000, 2001; Krzywkowski and Nilsson 2017).

In the present report we characterized RNA template-dependent 3′-ribonucleotide/5′-DNA nick sealing efficiency and fidelity for PBCV-1 DNA ligase and T4Rnl2. The observed increase of ligation efficiency of chimeras for both enzymes on RNA motivated us to evaluate if iLock probe assay sensitivity can also be improved. We studied the effect of various ribonucleotide substitutions on the iLock probe activation and ligation by amplifying and digitally quantifying chimeric ligation products using rolling circle amplification (Jarvius et al. 2006; Krzywkowski et al. 2018). This was possible due to the ability of phi29 DNA polymerase to accept and reverse transcribe a limited number of ribonucleotides in chimeric circular templates, as recently described (Krzywkowski et al. 2018). We provide a systematic evaluation of ligation fidelities with chimeric and nonchimeric padlock and iLock probes for both PBCV-1 DNA and T4Rnl2 and demonstrate their potential for application in the detection and discrimination of miRNA in vitro and mRNA in situ.

## RESULTS

### Fidelity and efficiency of RNA-templated chimeric strand joining by PBCV-1 DNA ligase and T4Rnl2

**FIGURE 1.**
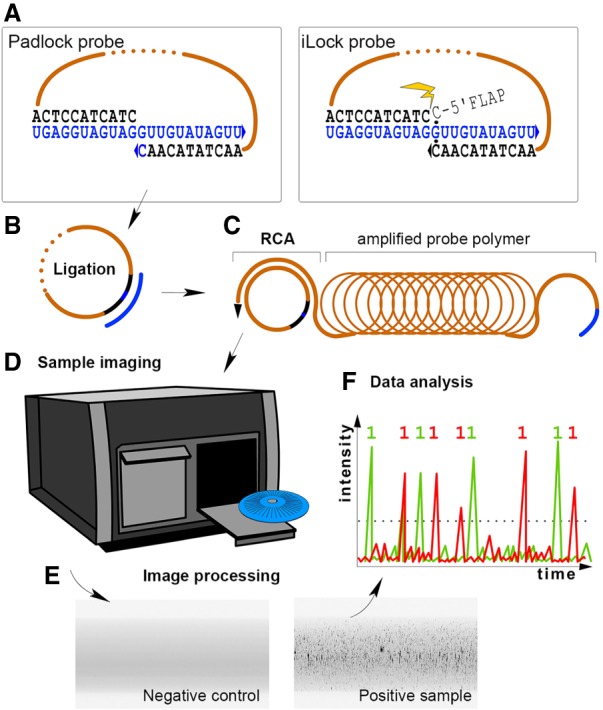
Experiment schematics. (*A*) Padlock or iLock probes are hybridized to RNA target (blue nucleotides). In iLock probes, noncomplementary extension on the 5′ end becomes removed by the polymerase (indicated with a spark). This step is described in detail in Krzywkowski and Nilsson (2017). Ligation probes contain a reporter within a sequence linking target-complementary arms (dotted). (*B*) Probes become ligated on RNA and target serves as primer for rolling circle amplification (RCA). (*C*) During RCA, a linear polymer of the probe copy is created. (*D*,*E*) Samples are fluorescently labeled using the reporter and imaged. (*F*) Labeled rolling circle amplification products—visible as bright speckles in the microfluidic channel—are quantified.

Our initial motivation was to investigate the unexplored activity of PBCV-1 DNA ligase to seal 3′ ribonucleotide -5′ DNA nicks templated by RNA. Since ribonucleotides on the 3′ acceptor terminus have been shown to improve ligation efficiencies of T4Rnl2 (Nandakumar et al. 2004; Bullard and Bowater 2006), we hypothesized that ribonucleotide substitutions might exert a similar effect when using PBCV-1 DNA ligase. In order to measure ligation efficiencies and fidelities we used a method that digitally quantifies individual probe ligation products by amplifying ligated probes in a rolling circle amplification (RCA) reaction, which generates concatenated single amplification products that are fluorescently labeled through hybridization of fluorophore-tagged complementary DNA oligonucleotides to the detection sequence within the RCA products (Fig. 1). Fluorescently labeled RCA products are then quantified by fluorescently imaging RCA products flowed through a microfluidic channel (Fig. 1D–F), and as described in detail in Jarvius et al. (2006) (and commercially available as Aquila400).

**FIGURE 2.**
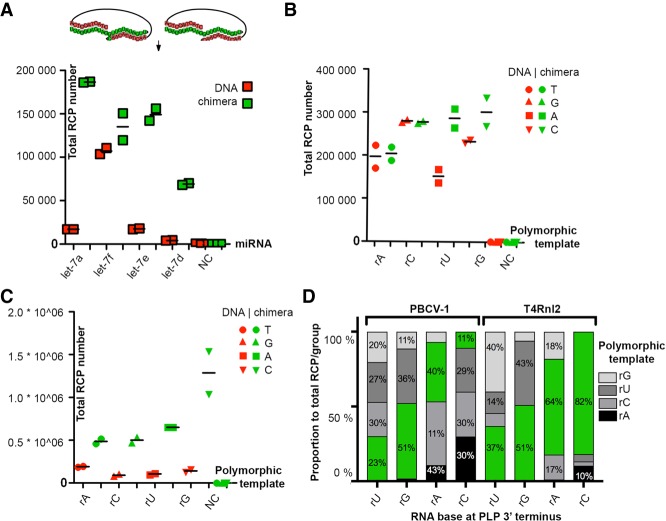
Effect of ribonucleotide substitutions on PBCV-1 DNA and T4Rnl2 RNA-dependent PLP ligation. (*A*) PLPs targeting four different *let-7* miRNA family members were designed with ribonucleotide (green) and DNA (red) terminal 3′ nucleotides. Probes were hybridized with corresponding miRNAs, ligated with PBCV-1, amplified and detected using microfluidic-based optical system as described in Materials and Methods. Total number of RCA products (RCP) for each PLP/miRNA pair is shown on *y*-axis while type of miRNA is depicted on *x*-axis. NC, negative control. (*B*,*C*) Comparison of DNA and 3′ ribonucleotide-modified PLP ligation efficiency for PBCV-1 and T4Rnl2. Full DNA (red) and chimeric (green) padlock probes were hybridized with a corresponding RNA and ligated with (*B*) PBCV-1 and (*C*) T4Rnl2. (*D*) Effect of 3′-OH(rN) mismatches on nick sealing fidelity for PBCV-1 and T4Rnl2. Numbers of RCPs for each RNA template were added and presented as percentage within a PLP group. Calculated proportion for the expected probe pair is highlighted in green.

Using synthetic *let-7* RNA templates, we compared the probe-end ligation efficiency of PBCV-1 DNA ligase on the usual DNA padlock probes (PLPs) with 5′ DNA and 3′ DNA ends, and chimeric PLPs with 5′ DNA end and 3′ ribonucleotide ends (Fig. 2A). We measured the ligation efficiency as total number of rolling circle amplification products (RCPs), digitally counted for each PLP - template pair. On synthetic miRNAs, chimeric PLPs showed higher ligation efficiency than pure DNA PLPs (Fig. 2A). We then aimed to systematically characterize the ligation efficiency and fidelity of chimeric PLPs on a panel of synthetic RNA templates with only a one base difference (A, G, C, or U) within the center of an otherwise identical template sequence, further on referred to as polymorphic templates (Fig. 2B–D; Supplemental Fig. 1).

For PBCV-1 DNA ligase, the ligation efficiency of chimeric PLPs was higher for 3′ rU PLPs on the A-template and 3′ rC PLPs on the G-template, while for the pairs rA/U and rG/C no significant difference in efficiency between chimeric and pure DNA PLPs was observed. For T4Rnl2, the effect of 3′ ribonucleotide modifications on ligation efficiency, on the other hand, was much stronger (Fig. 2C). As expected, T4Rnl2 readily ligated chimeric PLPs, while relatively lower activity was seen for DNA PLPs (Fig. 2C). Overall, ligation efficiency for probes with 3′ ribonucleotide modifications was improved for PBCV-1 DNA ligase, however, not to the same extent as on the much shorter miRNA targets (Fig. 2A vs. 2B). For T4Rnl2, a strong increase (approximately three- to fourfold) of ligation efficiency was observed (Fig. 2C).

Furthermore, we investigated the fidelity of both ligases on chimeric and pure DNA PLPs using a single base mismatch model. To study the effect of single-base mismatches of PBCV-1 DNA ligase and T4Rnl2 end-joining activity, four chimeric PLPs (differing with a terminal 3′ nucleotide rU, rG, rA, rC, Supplemental Table 1), were individually hybridized with RNA polymorphic templates, ligated and amplified with RCA (Fig. 2D). As expected, PBCV-1 DNA ligase catalyzed efficient ligation of 3′(rN)/5′(N), but with low sequence fidelity. PBCV-1 DNA ligase was highly tolerant toward most 3′ ribonucleotide mismatch combinations (Fig. 2D; Supplemental Fig. 1A). In line with previous observations (Sriskanda and Shuman 1998; Krzywkowski and Nilsson 2017), 3′-OH rG and rC represented most substrate-specific configurations. T4Rnl2 on the other hand, was moderately accurate ligating rC/rG (82%) and rA/rC (64%) but showed poor end-joining fidelity toward other combinations (Fig. 2D; Supplemental Fig. 1B). The ligation rate of nonchimeric PLPs was 10%–40%, in comparison to chimeric substrates, depending on the base ligated (Fig. 2C).

### Effect of various ribonucleotide substitutions on RNA templated iLock probe activation and PBCV-1 ligation efficiency

**FIGURE 3.**
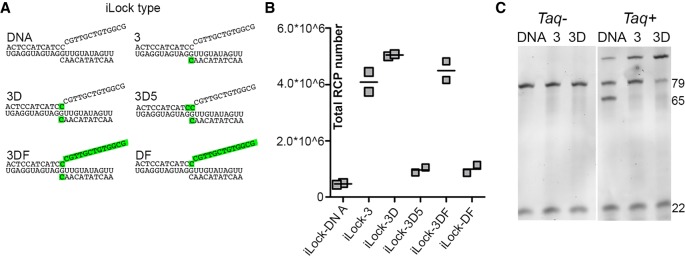
Effect of various ribonucleotide substitutions on iLock probe RNA detection assay with PBCV-1 ligase. Recognition of the invader structure and structure-specific endonucleolytic activity of *Taq* DNA polymerase can vary for different RNA substitutions. (*A*) Targeting *let-7a* with iLock probe. Ribonucleotides were introduced: at a terminal 3′ base (3); base in the 5′ arm, that an invading 3′ arm competes with for target binding (displaced base, 3D); base in the 5′ arm, that becomes a 5′-phosphorylated donor after iLock probe activation (3D5); in the flap sequence (3DF/DF). (*B*) iLocks were modified according to *A*, except nonchimeric iLock (DNA). The total number of RCPs for each probe is shown on *y*-axis. (*C*) PAGE of three selected iLock probes (DNA, 3, 3D) after activation and ligation, without (first three lanes) and with *Taq* DNA polymerase (last three lanes). Nonactivated iLock probe (79) is shortened upon activation by 14 nt (65) and ligated (seen as high molecular weight band at the *top* of the gel). (22) *let-7a* miRNA.

In order to improve end-joining fidelity on RNA templates and to allow for single base discrimination on RNA, another recently described probing scheme—invader PLPs (iLocks)—can be used. In this scheme, we utilized structure-specific 5′ flap cleavage activity of *Taq* DNA polymerase, to activate properly annealed iLock/RNA pairs for ligation (Krzywkowski and Nilsson 2017). As PBCV-1 DNA ligase tolerated the majority of chimeric 3′ mismatches, we aimed to explore whether the iLock assay also increases sequence discrimination specificity using chimeric probes. For this purpose, we tested how presence of ribonucleotide substitutions in various positions of an iLock probe affects probe activation and ligation, compared to a pure DNA iLock probe. Multiple iLock probes targeting *let-7a* miRNA were designed (Fig. 3A; Supplemental Table 2), containing ribonucleotide or RNA substitutions in various probe positions. The chimeric “iLock-3” carried a ribonucleotide substitution at the 3′ terminus. The “iLock-3D” carried the same terminal 3′ ribonucleotide and additionally one ribonucleotide substitution at the displaced base of the 5′ flap. The “iLock-3D5” probe carried an additional ribonucleotide base at the position 5′ from “iLock-D” RNA. This ribonucleotide base would be positioned at the 5′-phosphate donor end after successful iLock activation. Lastly, “iLock-3DF” carried the terminal 3′ and the complete 5′ flap as RNA, and “iLock-DF” carried only the 5′ flap with RNA with no 3′ terminal ribonucleotide substitution.

Compared to unmodified iLock, chimeric iLock-3 demonstrated a remarkably increased activity on *let-7a* miRNA (Fig. 3B,C). According to PAGE of the activated and ligated iLock probes (Fig. 3C), only a fraction of nonchimeric iLocks were activated at given assay conditions (well 4, visible as strong 65 bases long band) and an even smaller fraction was successfully ligated (faint band in the upper part of the gel). In contrast, essentially all activated iLock-3 probes became ligated as evident by a quantitative gel shift in Figure 3C (well 5, top band), also reflected by the total number of RCA products generated with iLock-3 probe (Fig. 3B). When the flap nucleotide displaced by the invading terminal 3′ ribonucleotide was also substituted with ribonucleotide, as in iLock-3D, an additional efficiency increase was observed. iLock-3D probes were activated and ligated with highest efficiency (Fig. 3C, well 6). Importantly, when the “D” base and the flap was fully composed of RNA (iLock-DF), the probe performance declined. When the terminal 3′ base was substituted with ribonucleotide (iLock-3DF), the high efficiency was restored (Fig. 3B). A significantly increased performance of chimeric iLock-3D probes compared to pure DNA iLock probes was also observed for other short RNA that were assayed (*mir-21*, *let-7f*), both using PBCV-1 and T4Rnl2 (Supplemental Fig. 3). Finally, previous studies showed that 3′(N)/5′(rN) substrates have no effect on the ligation rate of PBCV-1 on DNA (Sriskanda and Shuman 1998). Contrary to this observation, we observed substantially reduced performance of iLock-3D5 probe on RNA (Fig. 3B). Collectively, our results clearly demonstrate that terminal 3′ and “D” ribonucleotide substitutions in the iLock probe enhance probe activation and ligation on RNA.

### Chimeric iLock RNA detection assay fidelity with PBCV-1 DNA ligase and T4Rnl2

**FIGURE 4.**
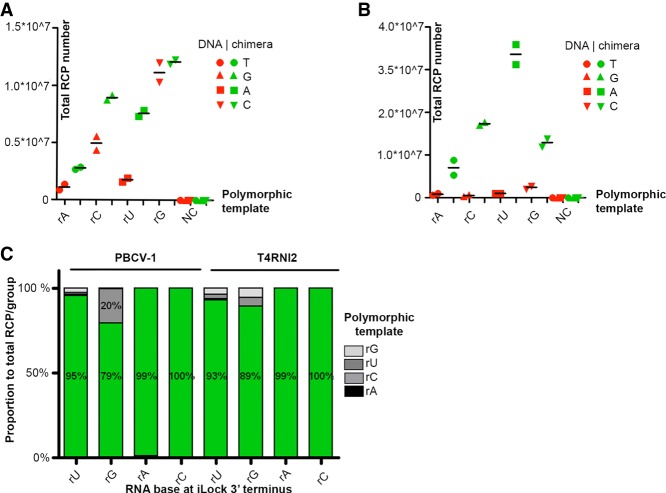
Effect of 3′-OH(rN) mismatches on iLock activation and nick sealing fidelity for PBCV-1 and T4Rnl2. (*A*,*B*) Chimeric iLock-3D (green) and nonchimeric (red) iLock probes performance on polymorphic RNA targets. Total number of RCPs for each probe on matching polymorphic templates is shown on *y*-axis for (*A*) PBCV-1 DNA ligase and (*B*) T4Rnl2. NC, negative control. (*C*) Fidelity of nick sealing by PBCV-1 DNA ligase (*left* panel) and T4Rnl2 (*right* panel) using 3D-type iLock probes on RNA. Numbers of RCPs for the same iLock probe on each RNA template were added and presented as percentage within an iLock probe group. Calculated proportion for the expected probe pair is highlighted in green.

To test if accuracy of RNA sensing with chimeric iLock-3D probes is maintained, we targeted the four polymorphic RNA templates (Supplemental Table 2) with four chimeric iLocks-3D probes. Consistent with our previous observations for chimeric PLPs, PBCV-1 ligation on longer RNA targets was improved using iLock-3D format (Fig. 4A). As expected, T4Rnl2 displayed greater ligation activity toward chimeric iLocks (Fig. 4B). Chimeric iLock probes showed excellent fidelity toward matching rA/rU, rU/rA and rC/rG probe pairs (Fig. 4C; Supplemental Fig. 4). The rG chimeric iLock probe—in contrast to G/rG iLock (Krzywkowski and Nilsson 2017)—was activated on nonmatching RNA templates, rU in particular. Although such activity was five-/20-fold lower for PBCV-1/T4Rnl2 when compared to matching iLock probes, repositioning of the activation site could be a strategy to minimize false positive iLock activation in single base discrimination assays, hence maximizing accuracy of RNA detection assays. Importantly, iLock-3D probes showed no ligation products in template negative control reactions (Fig. 4A,B).

### Differentiation of conserved mRNAs in situ using chimeric PLP and iLock probes in mouse and human fibroblasts

**FIGURE 5.**
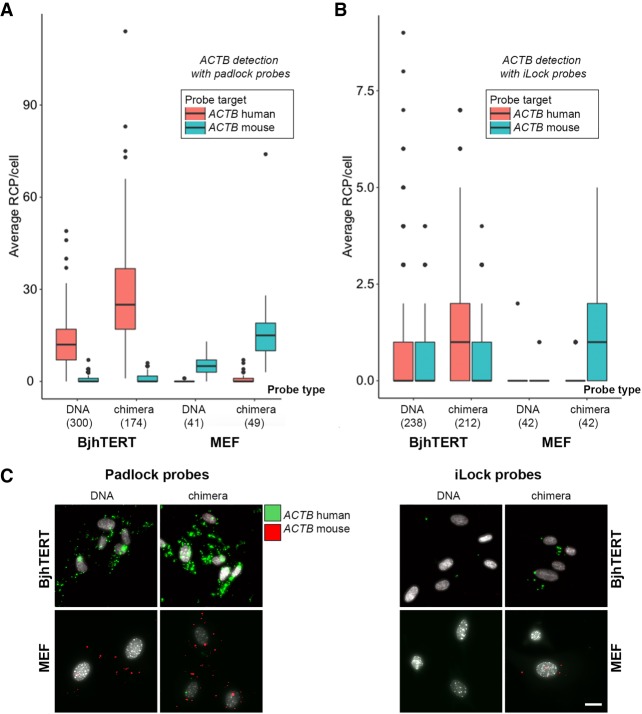
*ACTB* detection in situ using chimeric/nonchimeric PLP and iLock probes in human and mouse cell lines. (*A*,*B*) Single cell *ACTB* quantification in human (BjhTERT) and mouse (MEF) fibroblasts using PLPs (*A*) and iLocks (*B*). Probes targeting both species were used in cells followed by imaging and image processing as described in Materials and Methods. The number of RCP/cell for both species-specific probes was automatically calculated for every cell and presented as box plot (red plot shows counts for human-specific and blue for mouse-specific probe). Character of probe used (DNA or chimeric), number of cells analyzed in the experiment as well as species imaged (BjhTERT or MEF) are presented in the *x*-axis. The *lower* and *upper* hinges of box plots correspond to the first and third quartiles (the 25th and 75th percentiles). Thicker line across box plot, median; *upper* whisker, highest value that is within 1.5× the interquartile range of the hinge; *lower* whisker, lowest value within 1.5× the interquartile range of the hinge. (*X*-axis) (****) *P* ≤ 0.0005; (*) *P* ≤ 0.05. (*C*) Representative images that were used to generate *A* and *B*. Scale bar, 20 µm.

Detection of mRNA molecules in situ using circularizable probes has multiple advantages, including less demanding optimization of probe design or hybridization conditions, as well as the ability to discriminate single nucleotide target variations (Krzywkowski and Nilsson 2018). PLPs are commonly used for in situ analysis, but target mRNA is frequently converted to cDNA prior to ligation to enable use of high-fidelity thermostable DNA ligases (Larsson et al. 2010; Ke et al. 2013). Furthermore, amplification of PLPs ligated directly on long target RNA (Banér et al. 1998) restricts detection of mRNA to poly(A) 3′ termini (Lagunavicius et al. 2009), or where cellular RNA was enzymatically fragmented (Merkiene et al. 2010). To test if ribonucleotide substitutions in padlock and iLock probes could also improve detection of RNA in situ, we targeted highly conserved *ACTB* mRNA 3′ termini in human and mouse cultured fibroblasts with both PLPs and iLock probes, in either chimeric or pure DNA form (Supplemental Fig. 5; Supplemental Table 3). To maximize detection specificity, target site was chosen such that a six bases long gap would effectively limit cross-reactivity between the two target-probe pairs (Supplemental Fig. 5). After probe ligation (or invader probe activation and ligation in the iLock probe assay) the ligated probe circles were amplified with RCA directly in situ. The RCA products were then fluorescently labeled and imaged using fluorescent microscopy. Image analysis was performed to quantify the RCA products and RCA products/cell was determined and compared between the different assay conditions. Sensitivity of mRNA detection was significantly increased for both chimeric padlock and chimeric iLock probes compared to pure DNA probes (Fig. 5). Due to careful selection of the target site (Supplemental Fig. 5), both chimeric PLPs and iLocks generated minimal background between human and mouse species, as only few false positive RCPs (MEF-specific RCPs in BjhTERT, and vice versa) were present in the imaged cells (Fig. 5). The efficiency of probe ligation on RNA using chimeric PLPs was approximately threefold higher than pure DNA PLPs (Fig. 5B) while the efficiency of chimeric iLocks was considerably lower compared to chimeric PLPs (Fig. 5A).

## DISCUSSION

This study shows that chimeric padlock and iLock probes can be used as more efficient substitutes for ligation-based direct RNA sensing. We systematically analyzed ligation efficiencies and fidelities for different probe–target nucleotide combinations. To measure the rate of nick sealing, circularized probes where amplified using rolling circle model (RCA) and digitally counted. We conclude that the enzyme properties presented in this work were attributed to the ligation step rather than biased amplification since single ribonucleotide substitution exerts no effect on phi29 DNA polymerization during RCA (Krzywkowski et al. 2018).

According to our observations, probes with single ribonucleotide bases localized on the acceptor 3′ end were generally preferred over their DNA equivalents. We have observed a high tolerance toward mismatches at the ligation nick for PBCV-1 DNA ligase and similar observations were previously made for DNA substrates (Krzywkowski and Nilsson 2017). Interestingly, ligation rate decreased when an additional ribonucleotide substitution was introduced at the 5′ end of the ligation nick (Fig. 3A). The suppressive effect of the 5′ ribonucleotide suggests that PBCV-1 ligase 5′-donor substrate must adopt a B-form helical conformation (or intermediate A–B in character) to successfully participate in the ligation. The hypothesis that PBCV-1 ligase is conformation-specific, not sequence-specific, is further supported by the highly efficient ligation of mismatched substrates (Fig. 2D).

In line with the literature (Nandakumar et al. 2004; Bullard and Bowater 2006), oligonucleotide acceptors with a 3′-OH ribonucleotide were readily ligated by T4Rnl2 and mismatch tolerance was slightly lower when compared to PBCV-1 DNA ligase (Fig. 2C,D). Interestingly, the common notion is that T4Rnl2 permits efficient RNA templated DNA ligation provided there are at least two ribonucleotide bases at 3′-OH acceptor end (Nandakumar and Shuman 2004). While 3′ terminal ribonucleotide was suggested to directly engage in a T4Rnl2 substrate recognition, a second ribose was inferred to participate in an effective substrate binding under conditions below saturating of either enzyme or a substrate (Nandakumar and Shuman 2004). According to our observations, a single ribonucleotide at the 3′ terminus was sufficient to support efficient ligation, possibly because T4Rnl2 was used in concentrations greatly exceeding ligated substrates.

In iLock RNA detection assay, substitution of two particular nucleotide positions with ribonucleotides led to a significantly increased probe ligation rate on RNA. We hypothesize that this can be explained by the activity of *Taq* DNA polymerase during DNA replication that mimics native cleavage of displaced RNA primers on the replicated lagging strand. T4Rnl2 compatibility with the iLock RNA detection assay demonstrates that this RNA sensing concept is not limited by ligase type and can be combined with any enzyme, especially if low ligation fidelity poses an experimental challenge. One (or two in iLock3D) ribonucleotide modifications are an inexpensive addition that substantially increases assay sensitivity without punishing RNA detection fidelity.

It is also worth highlighting that for both padlock and iLock probes, the ribonucleotide effect was higher when substrates were ligated on short RNA templates for both ligases included in the study. In addition to influencing enzyme kinetics, ribonucleotide bases can also contribute to greater nucleic acid duplexes stability (Elliott and Ladomery 2011). More stable duplexes would ligate faster during the initial reaction stage. However, our assay format did not allow detection of really fast ligation kinetics. Both chimeric and nonchimeric PLPs generated similar data during early reaction stages (Supplemental Fig. 2B,C). When ligase concentration was titrated, we observed a proportional decrease in RCP number, not recovered by prolonged ligation. These observations suggest that the PBCV-1 DNA ligase is not turned over during the RNA templated reaction. The reaction only reached saturation if an excess of enzyme over substrate was used (Supplemental Fig. 2A). Similar observations have previously been made for RNA templated DNA ligation using T4 DNA ligase (Nilsson et al. 2001).

In cells fixed on slides, we showed that chimeric padlock and iLock probes can be applied to discriminate highly similar mRNA sequences. Compared to our in vitro observations however, overall activity of chimeric iLocks in situ was marginal. This was most likely due to suboptimal iLock activation conditions for thermostable *Taq* DNA polymerase. A reaction temperature of 37°C was preferential for activity of PBCV-1 DNA ligase as well as for hybridization of short target-complementary probe arms. Chimeric padlock and iLock probes, in combination with RCA, provide a promising tool set to detect and discriminate with high specificity between highly similar miRNA and mRNA sequences in vitro and in situ, i.e., in preserved cells and tissues. While readily applicable for in vitro applications, iLock assays in situ require further protocol optimization.

## MATERIALS AND METHODS

### Oligonucleotides used in the study

All oligonucleotides used in the present work were purchased from IDT (Integrated DNA Technologies, Inc.) using the following synthesis and purification conditions: DNA PLPs and iLock probes: 4 nM of standard desalted Ultramer DNA oligonucleotides; chimeric padlock and iLock probes: 4 nM of standard desalted Ultramer RNA oligonucleotides; decorator probes: HPLC purified DNA oligonucleotides with 5′ conjugated fluorophore. All PLPs were prephosphorylated on the 5′ terminus to permit ligation. RNA templates harboring a centrally located polymorphic site used in the study were described previously (Krzywkowski and Nilsson 2017). Probes were designed, such that terminal arms would form a nicked circle when base paired with attended RNA targets and a discriminatory base was localized at the 3′ terminus of the probe (Supplemental Table 1). miRNA PLPs used in this study were described previously (Supplemental Table 1; Krzywkowski and Nilsson 2017). Chimeric PLPs were ordered with a terminal 3′-OH ribonucleotide (Supplemental Table 1). iLock probes, used in the present work for comparative purposes, were described previously (Krzywkowski and Nilsson 2017; Supplemental Table 2). The standardized chimeric iLock probes design includes ribonucleotide substitution on the terminal 3′ base as well as a base in the 5′ arm that the 3′ terminal base was competing for target binding with (displaced base, Fig. 2A; Supplemental Table 2). For traditional rolling circle product (RCP) staining and digital quantitation, a reporter sequence was embedded in the sequence linking PLPs arms, separated from probe arms with a series of 10 adenines.

### RNA detection assay and digital quantitation of amplified iLock and padlock probes

iLock activation was performed in a 4:1 probe to template excess (typically, a 2 nM iLock probe was mixed with 0.5 nM RNA template). Duplicate reactions were incubated in a heated-lid thermocycler at 51°C for 30 min, in a 10 µL volume containing 0.1 U/µL of Taq DNA polymerase (ThermoFisher Scientific), 0.4 U/µL U RNase Inhibitor (Blirt) and 1× *Taq* polymerase buffer supplied with 8 mM MgCl2. Next, 3 µL of sample volume was transferred to a ligation reaction mix supplemented with 0.25 U/µL of PBCV-1 DNA ligase (SplintR, M0375S, NEB) or 0.2 U/µL of T4Rnl2 (M0239S, NEB) in respective buffers in a final volume of 15 µL. The reactions were incubated at 37°C for 30 min. For PLPs, identical ligation conditions were applied, excluding the activation step. RCA was conducted as described previously (Krzywkowski and Nilsson 2017) and a theoretical, final concentration of amplified products was 5 pM and 20 pM for padlock and iLock probes, respectively. Fifteen microliters of the RCA sample were analyzed using the Aquila 400 Detection Unit (Q-linea). If RCP concentration was outside the instrument's dynamic range, samples were diluted in 4 nM of the decorator probe in 1× labeling solution (20 mM EDTA, 20 mM Tris-HCl [pH 7.5], 0.05% Tween 20 and 1 M NaCl), incubated at 65°C for 3 min, allowed to cool at room temperature for 15 min and recounted. Template-negative reactions were run in parallel with every experiment as a control.

### In situ RNA detection

BjhTERT and MEF cells were cultured and fixed as described previously (Krzywkowski and Nilsson 2018). Fifty microliter volume secure seal chambers (Sigma–Aldrich) were applied on SuperFrost Plus slides (ThermoFisher Scientific), in four-on-each-slide format. Cells were rehydrated with DEPC-PBS prior to the experiment. 0.1 µM final concentration DNA/chimeric padlock/iLock probes were prehybridized in cells (human and mouse specific probes were designed with unique decorator sequences to facilitate simultaneous discrimination, Supplemental Table 3) in hybridization buffer (0.475 M Tris-HCl pH 8, 0.95 mM EDT, 0.76 M NaCl), supplemented with 0.8 U/µL RNase inhibitor for 2 h at 37°C. Each reaction step was followed by two washes with prewarmed DEPC-PBS-Tween 0.05%. Probe ligation was conducted as for in vitro reactions with the following differences: probes were omitted from the reaction; reaction was conducted at 37°C for 2 h; 0.1 U/µL *Taq* DNA polymerase was additionally supplemented in reactions where iLock probes were used and incubated at the same conditions as for ligation alone; 0.8 U/µL RNase Inhibitor was supplemented in all reactions. RCA (37°C for 6 h), hybridization of decorator oligonucleotides, nuclei counterstaining and mounting of slides was described previously (Krzywkowski and Nilsson 2018). Slides were imaged with an Axioplan II epifluorescence microscope (Zeiss) equipped with an Orca Flash 4.0 v2 camera (Hamamatsu) with a total magnification of 200× using ZEN software. Stacks were superimposed using maximum intensity projection (MIP) in ZEN and exported as original black- and-white (BW) pictures for processing, as described elsewhere (Punga et al. 2018). Single cell data was exported as a .csv file and processed in RStudio (0.98.1091). In total, 300/174 BjhTERT cells were processed after treatment with DNA/chimeric PLPs and 41/49 MEF cells. 238/212 BjhTERT cells were processed after treatment with DNA/chimeric iLock probes and 42/42 MEF cells. Because the number of RCPs/cell for iLock probes was not normally distributed in the cells, a Wilcoxon signed-rank test was applied to compare chimeric and nonchimeric probes. *P*-values lower than 0.05 were considered statistically significant.

### PAGE

To visualize activation and ligation efficiency of various chimeric iLock probes (Supplemental Table 2), products were separated electrophoretically, as described previously (Krzywkowski and Nilsson 2017). In chimeric probes binding assay (Supplemental Fig. 2), concentrations of template and PLPs as stated above were incubated with 62 mU/ µL PBCV-1 DNA ligase and 0.4 U/µL RNase Inhibitor at room temperature for 10 min. Reactions were stopped by adding 1 µL 0.5 M EDTA and 5 µL 100% formamide (Sriskanda and Shuman 1998). PAGE was conducted as described above.

## SUPPLEMENTAL MATERIAL

Supplemental material is available for this article.

## Supplementary Material

Supplemental Material
