# Supplementary material for: Chimeric padlock and iLock probes for increased efficiency of targeted RNA detection
Source: RNA. 2019 Jan;25(1):82–9. doi: 10.1261/rna.066753.118 (PMC6298565; doi:10.1261/rna.066753.118)
Supplement: Supplemental Material [file supp_25_1_82__index.html]

Chimeric padlock and iLock probes for increased efficiency of targeted RNA detection — Supplemental Material 

# Chimeric padlock and iLock probes for increased efficiency of targeted RNA detection

## Supplemental Material

- Supplemental\_Material.docx
