# Supplementary material for: Chimeric padlock and iLock probes for increased efficiency of targeted RNA detection
Source: RNA. 2019 Jan;25(1):82–9. doi: 10.1261/rna.066753.118 (PMC6298565; doi:10.1261/rna.066753.118)
Supplement: Supplemental Material [file supp_066753.118_Supplemental_Material.docx]

**Supplementary table 1.** Oligonucleotide sequences used in experiment

*ID* *5’ modification* *Sequence (5’- 3’)*

hsa_let-7a rUrGrArGrGrUrArGrUrArGrGrUrUrGrUrArUrArGrUrU

hsa_let-7f rUrGrArGrGrUrArGrUrArGrArUrUrGrUrArUrArGrUrU

hsa_let-7e rUrGrArGrGrUrArGrGrArGrGrUrUrGrUrArUrArGrUrU

hsa_let-7d rArGrArGrGrUrArGrUrArGrGrUrUrGrCrArUrArGrUrU

let7-a_PLP_1 Phos CTACTACCTCA(10A)*CCTCAATGCACATGTTTGGCTCC*(10A)AACTATACAAC

let7-f_PLP_1 Phos CTACTACCTCA(10A)*CCTCAATGCACATGTTTGGCTCC*(10A)AACTATACAAT

let7-e_PLP_1 Phos CTCCTACCTCA(10A)*CCTCAATGCACATGTTTGGCTCC*(10A)AACTATACAAC

let7-d_PLP_1 Phos CTACTACCTCT(10A)*CCTCAATGCACATGTTTGGCTCC*(10A)AACTATGCAAC

let7-a_PLP_**RNA**_1 Phos CTACTACCTCA(10A)*CCTCAATGCACATGTTTGGCTCC*(10A)AACTATACAArC

let7-f_PLP**_RNA**_1 Phos CTACTACCTCA(10A)*CCTCAATGCACATGTTTGGCTCC*(10A)AACTATACAArU

let7-e_PLP_**RNA**_1 Phos CTCCTACCTCA(10A)*CCTCAATGCACATGTTTGGCTCC*(10A)AACTATACAArC

let7-d_PLP_**RNA**_1 Phos CTACTACCTCT(10A)*CCTCAATGCACATGTTTGGCTCC*(10A)AACTATGCAArC

plymorph_templ_C rUrCrUrCrGrCrUrGrUrCrArU*rCrCrCrUrArUrArUrCrCrUrCrG

plymorph_templ_A rUrCrUrCrGrCrUrGrUrCrArU*rArCrCrUrArUrArUrCrCrUrCrG

plymorph_templ_G rUrCrUrCrGrCrUrGrUrCrArU*rGrCrCrUrArUrArUrCrCrUrCrG

plymorph_templ_U rUrCrUrCrGrCrUrGrUrCrArU*rUrCrCrUrArUrArUrCrCrUrCrG

3’T_PLP_2 Phos ATGACAGCGAGA(10A)*AGTAGCCGTGACTATCGACT*(10A)CGAGGATATAGGT

3’G_PLP_2 Phos ATGACAGCGAGA(10A)*AGTAGCCGTGACTATCGACT*(10A)CGAGGATATAGGG

3’A_PLP_2 Phos ATGACAGCGAGA(10A)*AGTAGCCGTGACTATCGACT*(10A)CGAGGATATAGGA

3’C_PLP_2 Phos ATGACAGCGAGA(10A)*AGTAGCCGTGACTATCGACT*(10A)CGAGGATATAGGC

3’rT_PLP_2 Phos ATGACAGCGAGA(10A)*AGTAGCCGTGACTATCGACT*(10A)CGAGGATATAGGrT

3’rG_PLP_2 Phos ATGACAGCGAGA(10A)*AGTAGCCGTGACTATCGACT*(10A)CGAGGATATAGGrG

3’rA_PLP_2 Phos ATGACAGCGAGA(10A)*AGTAGCCGTGACTATCGACT*(10A)CGAGGATATAGGrA

3’rC_PLP_2 Phos ATGACAGCGAGA(10A)*AGTAGCCGTGACTATCGACT*(10A)CGAGGATATAGGrC

Decorator probe_1 Cy3 *CCTCAATGCACATGTTTGGCTCC^a^*Decorator probe_2 Cy3 *AGTAGCCGTGACTATCGACT^a^*

*r[N]: RNA oligonucleotide; (10A): linker; ^a^: last four bases of the decorator probe were 2’ O-methylRNA to prevent oligo hydrolysis by the phi29 polymerase; italics: decorator sequence. Identity of a decorator probe used for RCP staining is depicted as number at the end of the padlock probe ID*

**Supplementary table 2.** List of probes used in iLock activation efficiency and fidelity experiment

*ID* *5’ modification* *Sequence (5’- 3’)*

hsa_let-7a rUrGrAGrGrUrArGrUrArGrGrUrUrGrUrArUrArGrUrU

let-7a_PLP Phos CTACTACCTCA(7A)*CCTCAATGCACATGTTTGGCTCC(7A)*AACTATACAAC

iLock_1 CGCGTGTCGTTGC**C**CTACTACCTCA(10A)*CCTCAATGCACATGTTTGGCTCC*(10A)AACTATACAA**C**

iLock-3_1 CGCGTGTCGTTGC**C**CTACTACCTCA(10A)*CCTCAATGCACATGTTTGGCTCC*(10A)AACTATACAA**rC**iLock-3D_1 CGCGTGTCGTTGCr**C**CTACTACCTCA(10A)*CCTCAATGCACATGTTTGGCTCC*(10A)AACTATACAA**rC**iLock-3D5_1 CGCGTGTCGTTGC**rC**rCTACTACCTCA(10A)*CCTCAATGCACATGTTTGGCTCC*(10A)AACTATACAA**rC**

iLock-3DF_1 rCrGrCrGrTrGrTrCrGrTrTrGrC**rC**CTACTACCTCA(10A)*CCTCAATGCACATGTTTGGCTCC*(10A)AACTATACAA**rC**iLock-DF_1 rCrGrCrGrTrGrTrCrGrTrTrGrCr**C**CTACTACCTCA(10A)*CCTCAATGCACATGTTTGGCTCC*(10A)AACTATACAA**C**

plymorph_templ_C rUrCrUrCrGrCrUrGrUrCrArU*rCrCrCrUrArUrArUrCrCrUrCrG

plymorph_templ_A rUrCrUrCrGrCrUrGrUrCrArU*rArCrCrUrArUrArUrCrCrUrCrG

plymorph_templ_G rUrCrUrCrGrCrUrGrUrCrArU*rGrCrCrUrArUrArUrCrCrUrCrG

plymorph_templ_U rUrCrUrCrGrCrUrGrUrCrArU*rUrCrCrUrArUrArUrCrCrUrCrG

3’T_*i*Lock_3 TATATCCCTATAT**T**ATGACAGCGAGA(10A)*AGTAGCCGTGACTATCGACT*(10A)CGAGGATATAGG**T**3’G_*i*Lock_3 TATATCCCTATAT**G**ATGACAGCGAGA(10A)*AGTAGCCGTGACTATCGACT*(10A)CGAGGATATAGG**G**3’A_*i*Lock_3 TATATCCCTATAT**A**ATGACAGCGAGA(10A)*AGTAGCCGTGACTATCGACT*(10A)CGAGGATATAGG**A**3’C_*i*Lock_3 TATATCCCTATAT**C**ATGACAGCGAGA(10A)*AGTAGCCGTGACTATCGACT*(10A)CGAGGATATAGG**C**3’U_*i*Lock_**RNA**_3 TATATCCCTATAT**rU**ATGACAGCGAGA(10A)*AGTAGCCGTGACTATCGACT*(10A)CGAGGATATAGG**rU**
3’G_*i*Lock_**RNA**_3 TATATCCCTATAT**rG**ATGACAGCGAGA(10A)*AGTAGCCGTGACTATCGACT*(10A)CGAGGATATAGG**rG**
3’A_*i*Lock _**RNA**_3 TATATCCCTATAT**rA**ATGACAGCGAGA(10A)*AGTAGCCGTGACTATCGACT*(10A)CGAGGATATAGG**rA**
3’C_*i*Lock _**RNA**_3 TATATCCCTATAT**rC**ATGACAGCGAGA(10A)*AGTAGCCGTGACTATCGACT*(10A)CGAGGATATAGG**rC**Decorator probe_1 Cy3 *CCTCAATGCACATGTTTGGCTCC^a^*

Decorator probe_3 Cy3 *AGTAGCCGTGACTATCGACT^a^
r[N]: RNA oligonucleotide; bolded, underlined: nucleotides participating in invader structure formation; ^a^: last four bases of the decorator probe were 2’ O-methylRNA to prevent oligo hydrolysis by the phi29 polymerase; (10A): polyandenine linker; italics: decorator sequence.
Identity of a decorator probe used for RCP staining is depicted as number at the end of the padlock probe ID*

*
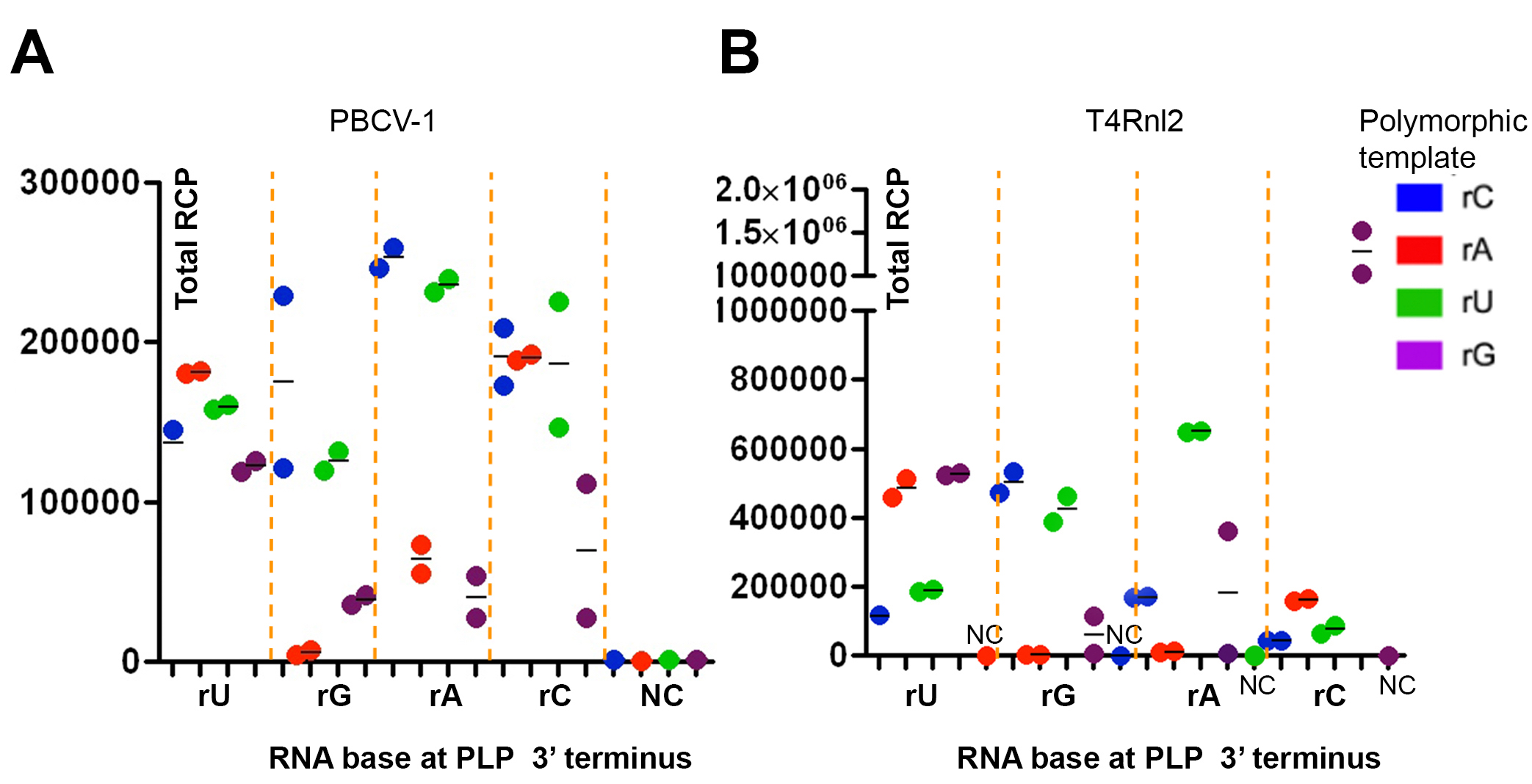
*

**Supplementary figure 1.** **PBCV-1 and T4Rnl2 ligase 3’-OH(rN)/5’-p(N) end joining fidelity on RNA**. A: The graphs show the data presented in the figure 1B as total counts of RCPs. PBCV-1 (A) and T4Rnl2 DNA (B) ligase. The y-axis shows the number of rolling circle products (RCPs) and the x-axis the padlock probe for which all four templates were used as ligation templates.


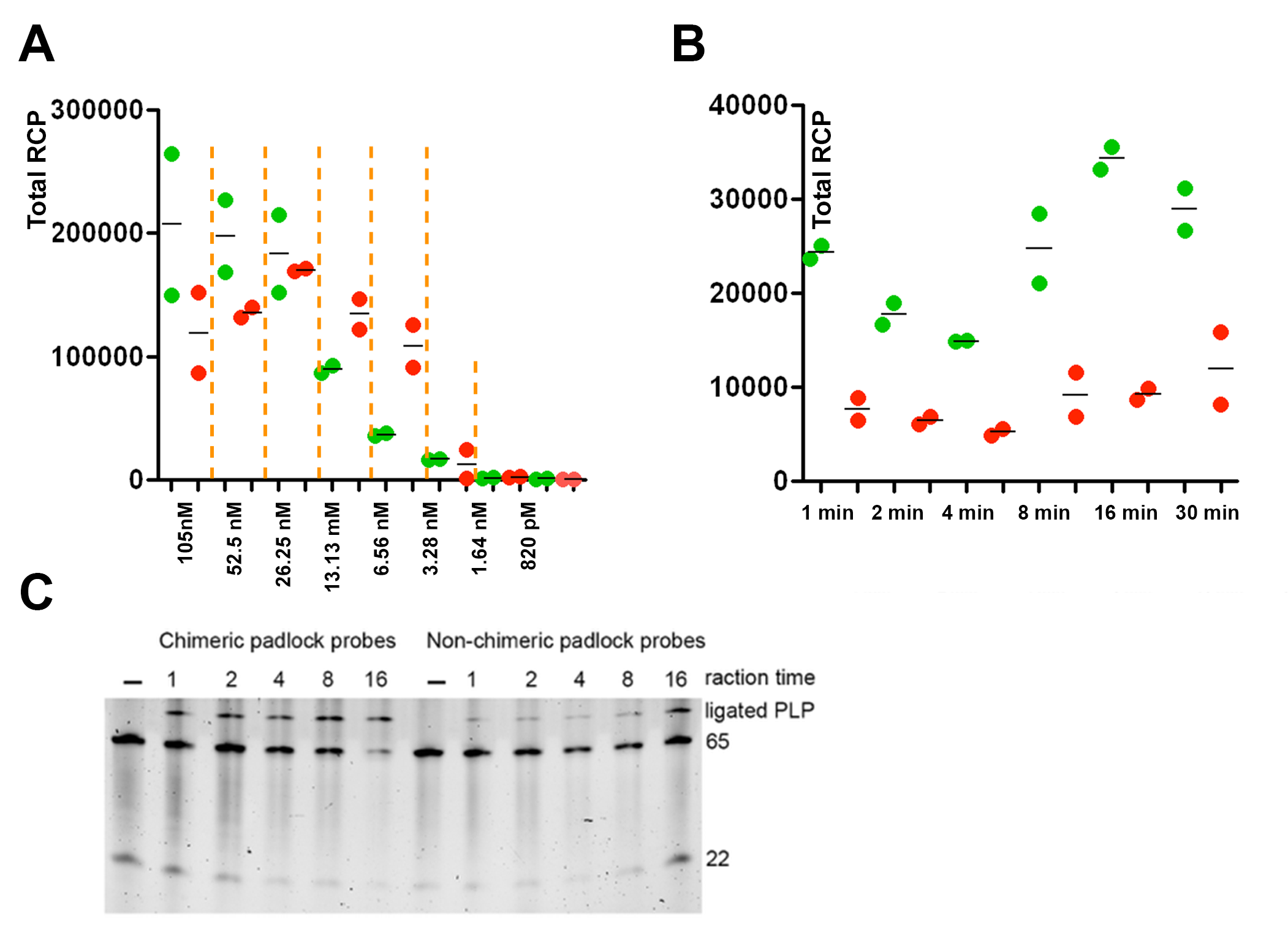


**Supplementary figure 2. Effect of RNA substitutions on 3’-OH(rG) and 3’-OH(G) padlock probe stability and ligation with PBCV-1 ligase on RNA.** A: PBCV-1 ligase titration. Total number of RCPs (y-axis) generated for each ligase concentration (x-axis) during 30 min ligation. Green points: chimeric probes; Red points: non-chimeric probes. To evaluate stability of chimeric padlock probes during first minutes of the reaction (B,C) 26.5 nM (62mU//μL) concentration was used. B: Ligation reaction was stopped by heat inactivating enzyme at 70°C for 10 min. Total number of RCA products (y-axis) for given time point (x-axis) is presented. C: Data from (B) but ligated products (not amplified) were resolved by PAGE as described in the materials and methods section. Ligation time elapsed is depicted as numbers above wells. –: Technical control where PBCV-1 DNA ligase was omitted. 65bp: non-ligated PLP; 22bp: RNA target.


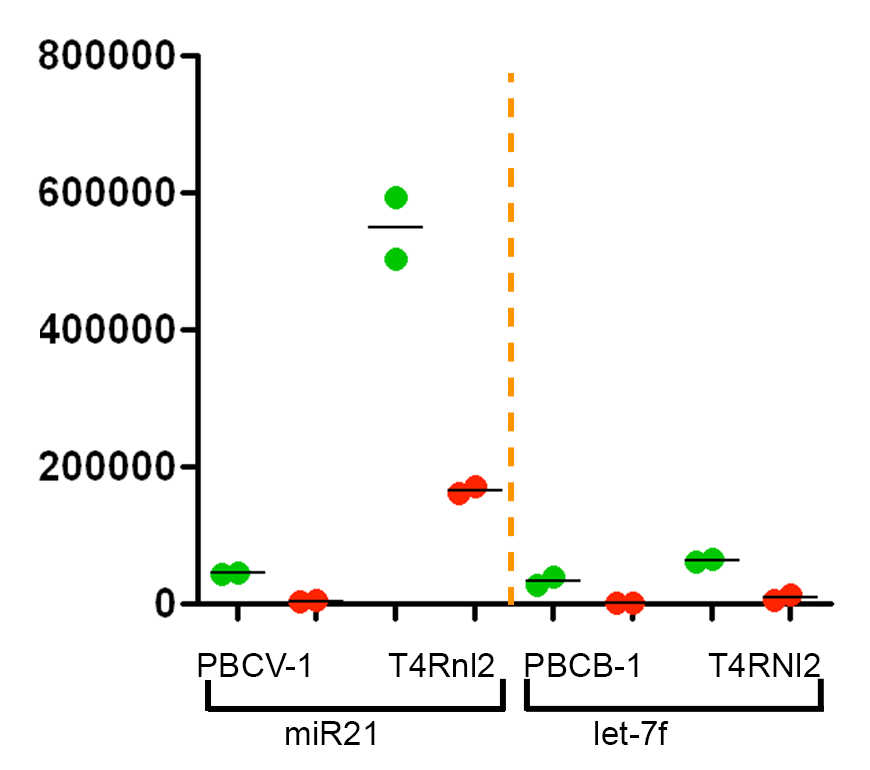


**Supplementary figure 3. Comparison of chimeric and non-chimeric iLock probes ligation on miR21 and let-7f using PBCV-1 and T4Rnl2.** Total number of RCPs for chimeric (green) or non-chimeric (red) iLock probes is presented on y-axis with PBCV-1 or T4Rnl2 (x-axis). Data is presented for miR21 and let-7f RNA template.


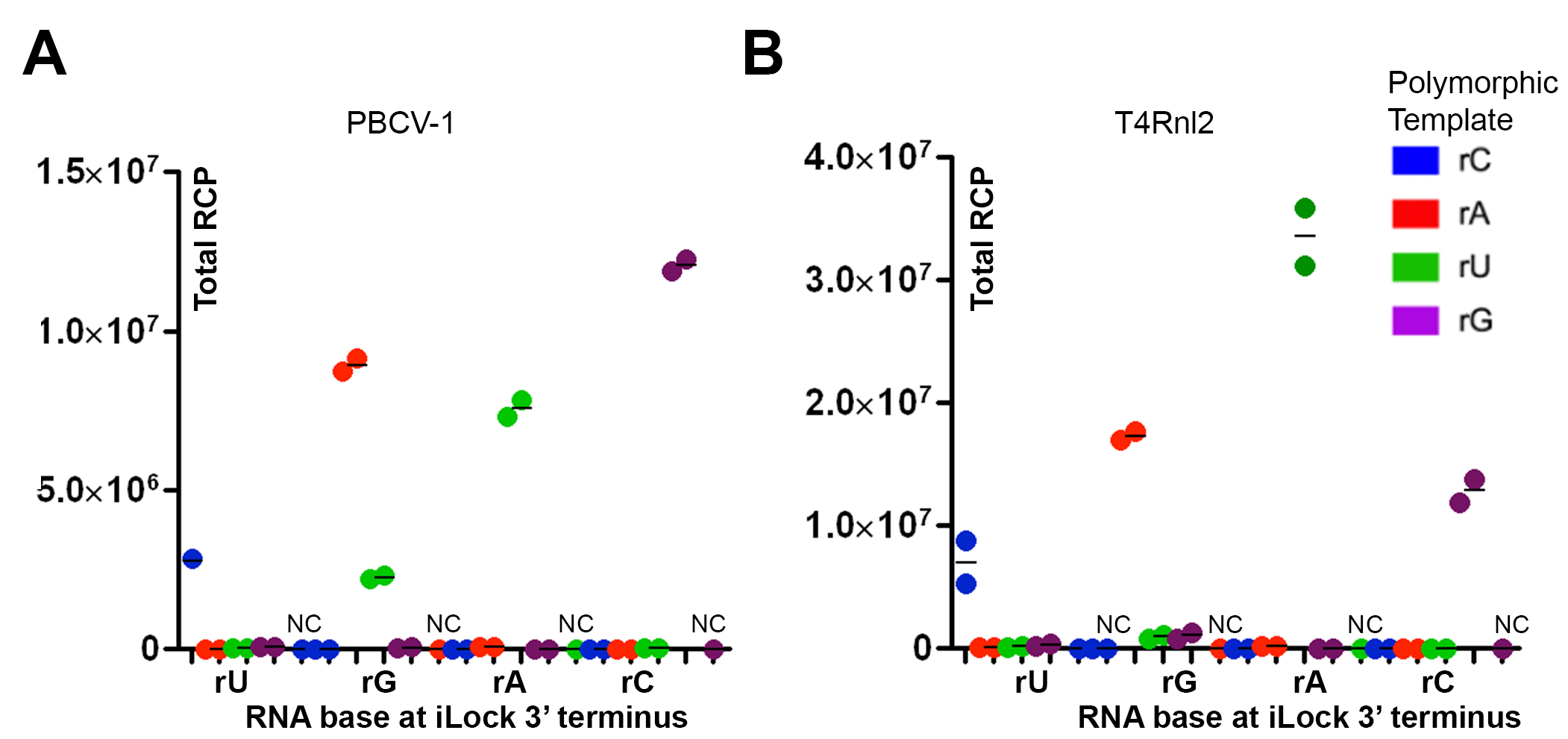


**Supplementary figure 4. PBCV-1 and T4Rnl2 ligase chimeric iLock probes ligation efficiency and fidelity on polymorphic templates**. Total number of RCPs generated and quantified (y-axis) for each iLock probe on each polymorphic template is shown for A) PBCV-1 DNA ligase and B) T4Rnl2.


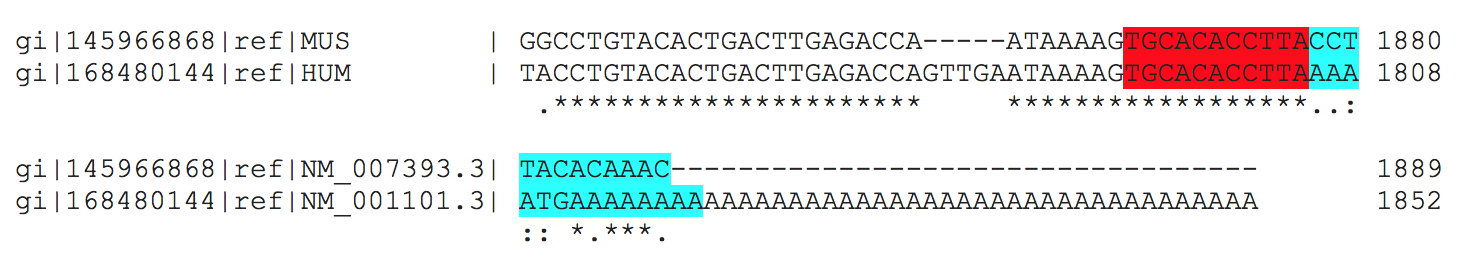


**Supplementary figure 5. ACTB conservation in mouse and human and selection of detected sequence.** Human and mouse ACTB mRNA sequence (accession numbers are given in the figure) were aligned and 3’ for both genes is depicted. To maximise specificity of probes, one probe arm was identical for both species (sequence highlighted in red) and one arm was chosen different (blue). Target regions and probe arms were identical for iLock and padlock probes.

**Supplementary table 3.** List of probes used in *ACTB* detection *in situ*

*ID* *5’ modification* *Sequence (5’- 3’)*

mmu_ACTB_PLP Phos TAAGGTGTGCA AAA(D1) AAA (D1) GTTTGTGTAAGG

hsa_ACTB_PLP Phos TAAGGTGTGCA (D2) AAA (D2) TTTTTTTTCATTTT

mmu_ACTB_PLP_chim Phos TAAGGTGTGCA AAA (D1) AAA (D1) GTTTGTGTAAGrG

hsa_ACTB_PLP_chim Phos TAAGGTGTGCA (D2) AAA (D2) TTTTTTTTCATTTrU
mmu_ACTB_iLock Phos TATATCCCTATAT **G** TAAGGTGTGCA AAA (D1) AAA (D1) GTTTGTGTAAG**G**
hsa_ACTB_iLock Phos TATATCCCTATAT **T** TAAGGTGTGCA (D2) AAA (D2) TTTTTTTTCATTT**T**

mmu_ACTB_iLock_chim Phos TATATCCCTATAT **rG** TAAGGTGTGCA AAA (D1) AAA (D1) GTTTGTGTAAG**rG**hsa_ACTB_iLock_chim Phos TATATCCCTATAT **rU** TAAGGTGTGCA (D2) AAA (D2) TTTTTTTTCATTT**rU**Decorator probe_1 (D1) Cy3 *AGTAGCCGTGACTATCGACT*

Decorator probe_2 (D2) Cy5 *AGTCGGAAGTACTACTCTCT
r[N]: RNA oligonucleotide; bolded, underlined: nucleotides participating in invader structure formation; (D1/2) decorator oligonucleotide sequence used as linker.*
